# “Helping fill that gap:” a qualitative study of aging in place after disaster through the lens of home-based care providers

**DOI:** 10.1186/s12877-021-02159-0

**Published:** 2021-04-08

**Authors:** Sue Anne Bell, Lydia K. Krienke, Sarah Dickey, Raymond G. De Vries

**Affiliations:** 1 University of Michigan School of Nursing, 400 North Ingalls Building, Ann Arbor, MI 48109 USA; 2 Johns Hopkins University School of Nursing, Baltimore, USA; 3 University of Michigan Medical School, Ann Arbor, USA

**Keywords:** Home-based care providers, Emergency preparedness, Disaster response, Natural disaster, Disaster management cycle

## Abstract

**Background:**

During a disaster, home-based care fills the critical need for continuation of health care. Home-based care is intended to function using existing care delivery models, continuing to provide care for patients wherever they are located, including in shelters and hotels. Home-based care providers are often the closest in contact with their patients —seeing them in place, even throughout a disaster— through which they develop a unique insight into aging in place during a disaster. The purpose of this study was to identify individual and community-level support needs of older adults after a disaster through the lens of home-based care providers.

**Methods:**

Using qualitative inquiry, five focus groups were conducted with home-based care providers (*n* = 25) who provided in-home care during Hurricane Irma and Hurricane Harvey. Participants were identified by contacting home health agencies listed in an open-source database of agencies participating in Centers for Medicare and Medicaid Services programs. Data were coded using an abductive analytic approach, and larger themes were generated in light of existing theory.

**Results:**

The results were distilled into eight themes that related to the importance of community and family, informal and formal supports throughout the disaster management cycle, maintaining autonomy during a disaster, and institutional and systemic barriers to obtaining assistance.

**Conclusions:**

In this study, home-based care providers described the challenges aging adults face in the response and recovery period after a large-scale disaster including maintaining continuity of care, encouraging individual preparedness, and accessing complex governmental support. Listening to home-based care providers offers new and important insights for developing interventions to address social and health needs for older adults aging in place after a large-scale disaster.

**Supplementary Information:**

The online version contains supplementary material available at 10.1186/s12877-021-02159-0.

## Background

In 2017, Hurricanes Irma and Harvey critically damaged coastal communities in the Southeastern United States, with Harvey alone causing $125 billion in damages [1, 2]. Events such as these are becoming more common as the effects of climate change advance. In fact, over 2600 federally-declared disasters have occurred since 2000 [3].

While we know anecdotally that older adults are at greater risk for adverse consequences from disasters, limited research exists to provide evidence of this effect. The consequences are hypothesized to include impacts on health, well-being, activities of daily living and support needs. Maintaining health and healthcare in a disrupted environment after a disaster can predispose chronically ill older adults, who have a need for access to regular and uninterrupted healthcare, to even greater morbidity [4].

Further, changes that come with aging can make navigating health and healthcare needs more challenging. Many older adults report needing support accessing and understanding healthcare systems and health insurance [5]. Older adults can face barriers to healthy aging at multiple levels during ‘normal’ times, from instrumental difficulties accessing healthcare at the policy level to decreased connections to social support networks. When normal community function is disrupted by a disaster, these barriers become more severe [6, 7].

An important channel for health care at the community level is home-based care. Home-based care providers remain in close contact with their patients—seeing them in place, even throughout a disaster—allowing for a unique insight at a level not available from other health care providers. By being present in patients’ homes, home-based care providers learn the ins and outs of their patients’ daily lives, schedules, and personal needs in ways that other healthcare providers do not [8, 9]. As well, time spent with patients in their own personal contexts exposes them to their patients’ individual struggles.

Home-based care services are intended to function using the existing care delivery structure in the event of a disaster—as evidenced in past disasters, where home health providers continued to care for their own patients in shelters and non-traditional healthcare facilities [10]. Past studies of home-based care and disaster have centered around home-based primary care in the U.S. Veterans Health Administration, [11–13] providing strong evidence for the success of disaster preparedness activities within a federally supported organizational structure.

**Fig. 1:**
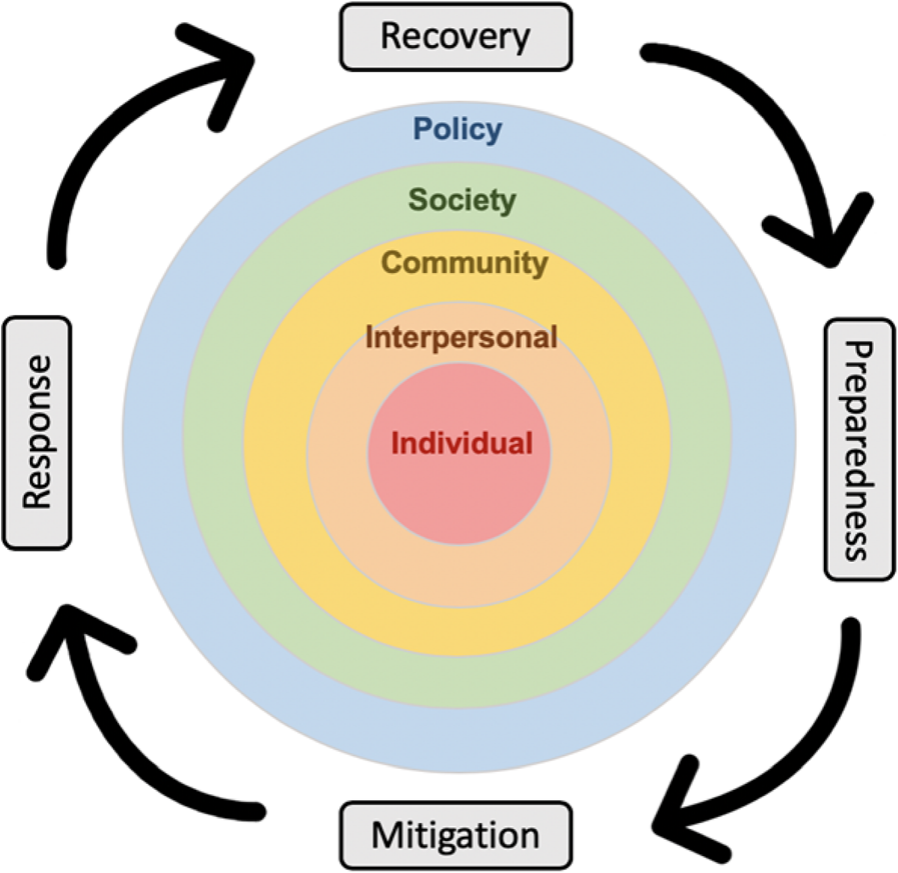
Social Ecological Model of Disaster Resilience

The Social Ecological Model of Disaster Resilience (SEMDR, Fig. 1), provides theoretical guidance for our study. This model, which incorporates the disaster management cycle of mitigation, preparedness, response, and recovery with the social ecological model [14] used for innovations in community health, can guide the understanding of healthy aging in the context of disaster. Social ecological models have been widely used in the health sciences as an organizing framework centered at the individual level, by situating the individual within the levels of influence that affect them. The model aids researchers in understanding individuals in the context of multiple levels of organization of the society they exist in in order to envision an environment conducive to meaningful change.

We apply the SEMDR to a set of qualitative interviews with home-based care providers who worked in areas affected by Hurricane Harvey and Hurricane Irma. The purpose of this study was to identify individual and community-level support needs of older adults after a disaster through the lens of home-based care providers. Because home-based care providers function as an integral part of the community, often residing in or close to the same areas as their patients, their perspectives can provide critical information on the challenges to, and strategies for, caring for older adults during disasters.

## Methods

This descriptive, qualitative study explored home-based care providers’ experiences providing care during a disaster. Institutional review board approval was received from the University of Michigan (HUM00132531). Focus groups were conducted in person in January, October, and November 2019. Participants provided written informed consent and were offered a $50 visa gift card via email for their participation.

### Interview guide

We developed an interview guide informed by prior conceptual work on disasters, home care and aging, and informed by the conceptual model in Fig. 1 [15–19]. The guide was refined through pilot testing with qualitative experts initially and then with a small group of registered nurses. The interview guide focused on barriers to health and healthcare access after the disaster, formal and informal community supports, strategies to support preparedness before the disaster, and strategies after the disaster to support safety and aging in place. The final interview guide was constructed for a goal interview length of 45 to 60 min (see guide in Supplement).

### Study sample and recruitment

Licensed healthcare providers who were employed by home health agencies in counties affected by Hurricane Harvey or Irma were included in this study. The study sample was derived using two sources: first, counties with Federal Emergency Management Agency (FEMA) individual assistance disaster declarations [3]. Individual assistance – support for individuals in need of assistance rebuilding homes and businesses – was conceptualized to be a useful metric of the extent to which a large-scale storm devastates community infrastructure. We prioritized home health agencies (HHA) operating within counties with individual assistance declarations. In order to capture the perspective from the home-based care workforce on caring for historically underserved patient populations, we took into consideration county demographic statistics on socioeconomic status and race. Counties with higher numbers of residents describing themselves as Black, Hispanic, Asian, and “other” were prioritized in the recruitment process, as well as counties with a median annual income closest to 2017 Federal poverty guidelines.

Our convenience sampling strategy recruited from home health agencies and started by contacting the 164 home health agencies in the counties that met the two criteria described above. All 164 agencies were contacted via telephone by a member of the study team, who explained the study to the agency administrator or their representative. If there was no answer or no one available to take the call, the agency was called back for a total of five times; after a fifth unanswered call or request for a call back, the agency was removed from the active calling sheet. Out of 164 agencies called, participants from five agencies attended focus groups. A sample size of 25 was targeted as we expected this number would provide adequate saturation of major themes [20, 21].

### Focus groups

Five focus groups were conducted in the two disaster-affected locations, Southern Florida (affected by Hurricane Irma in September 2017) and the greater Houston area (affected by Hurricane Harvey in August 2017), where data were generated from the perspective of the home-based care provider. The focus groups were held in-person in a private room at the participants’ place of employment. Each session was moderated by the principal investigator (SAB) who has doctoral-level training in qualitative methods, and a trained research assistant (LKK) who functioned as a secondary moderator. Participants had no prior interaction with the moderators. The secondary moderator took notes, managed audio recordings, and ensured coverage of themes through additional or clarifying probes. Focus group sessions lasted approximately 60 min and were guided by the same semi-structured questions and prompts (see Appendix)**.** Each session began with an introduction, which included a description of the study and the interviewer’s interest in the topic, and encouraged a comfortable environment for sharing thoughts and experiences as well as ensuring that all participants had an opportunity to speak throughout the session.

### Analysis

Data was analyzed using an abductive analytic approach [22], a method for analyzing data that combines inductive and deductive approaches allowing purposeful examination of a range of explanations, thereby reducing the likelihood of bias. The focus group conversations were recorded digitally and transcribed by a secure professional transcription service. After removing identifying information from the transcripts – including the mention of a specific facility, other clinician, or family member – the transcripts were formatted for coding. Using an iterative process, two research assistants independently generated codes in light of existing theory in keeping with abductive analysis, including the SEMDR framework.

Coders met face-to-face weekly in order to review and arbitrate differences in each other’s codes. The final 49 codes and their agreed upon definitions were then entered into a codebook in Microsoft Excel. After a systematic discussion and deliberation between all members of the study team, these codes were distilled into eight themes that represented larger over-arching concepts extracted from the focus groups transcript data. Using an abductive approach and the SEMDR, we mapped each theme to the level of the SEMDR. For example “*informal support within the community”* was mapped to the interpersonal level and response. These themes represent common strategies used by participants and the barriers they faced in providing adequate care for older adults during disasters.

## Results

### Demographics

Five focus groups were conducted with between two and six participants in each group. The five focus groups included a total of 25 home-based care providers. Of those that reported their occupation (80% of participants), 65% were Registered Nurses (RN) and 20% were Licensed Vocational Nurses (LVN). Of those that responded to the demographic survey [22], 12% were male and 88% were female. The majority of participants (84%) identified as White or Caucasian, 12% identified as Hispanic, and 4% as Black. The average length of time participants had been in their profession at the time of the focus group was 16.5 years, ranging from 3.5 to 39 years.

### Themes

We identified eight themes (see Table 1).

1. *The role of informal support within the community.*

**Table 1:** Organizing Themes

The role of informal support within the community	
Challenges in accessing disaster-related services and information for aging adults	
Socioeconomic factors as a determinant of recovery	
Role of family in caregiving	
Equating preparedness with previous hurricane experience	
Institutional and systemic barriers to preparedness	
Loss of material possessions represented loss of identity	
Disaster-related mental health care needs	

Participants described community support from institutions such as schools and faith-based organizations as leaders in the recovery effort. They provided gathering spaces and ready-made teams of people, such as members of local football teams and church congregations, to take on recovery activities such as mowing lawns and clearing debris. One participant described these informal supports:

I feel like it was much more of a community effort than it was an organized effort … And it was just anybody and everybody that could did, and they did whatever needed to be done. People took people in, strangers. I don't know you, but I have two bedrooms. You can come stay at my house till we figure out what's going on. Large groups of people just going through neighborhoods and literally mucking out, starting at one end and just mucking out and taking out furniture and things like that (B2).

Participants noted that older adults trusted their communities rather than governmental agencies as a primary source of support. Local organizations were described as being able to tailor their effort to the needs and values of the community. According to one participant,

At the end of the day, it was a community, helping community. They realize that government was not going to be their solution (E5).

Conversely, government agencies and insurers were seen as lacking in their efforts to supplement individual-level disaster support and community response. Participants reported that numerous barriers existed to applying for financial assistance for their older adult patients, largely because the application processes were challenging to understand and there was a lack of follow-through by the disaster aid agencies even after applications were completed.

I did have several patients that had to deal with FEMA … but they would just laugh about it, not really laugh about it but it's not ever going to happen because they couldn't figure out how to do it correctly or it's too big of a hassle to do it. They couldn't even do it. Their families were working on it or a daughter or son or somebody was working on it and even they were having trouble (E4).

2) *Challenges in accessing disaster-related services and information for aging adults*

Providers reported that the hurricanes more severely affected those who rely more on supportive services, especially those older adults with disabilities or health conditions and those with lower socioeconomic status. Participants used evacuation efforts as an example. Aging adults and those with mobility issues required additional time, resources, and planning measures to successfully evacuate. Participants acknowledged that many of their patients did not evacuate due to a lack of alternative housing options, an inability to pay for temporary lodging and discomfort with evacuation shelters. In addition, they reported that community evacuation assistance programs were not always available to those living in rural areas.

When you evacuated for a hurricane, you're on your own and you have to pay for it … So then they're stuck going wherever they can get a shelter to take them and stay in the shelter for a while (D4).

The only problem I think I've heard about that though is that you have to meet at a designated area and a lot of our homebound patients, especially if they live alone. I remember talking with one gentlemen about 2-1-1 and he's like, "Well, why? Because I live out in the middle of nowhere and I don't have the capability of getting to the designated area for them to pick me up" (E3).

Receiving information and accessing essential services presented additional challenges that posed barriers for aging adults. For example, participants reported that patients had to wait in long lines to apply at service centers, which disadvantaged aging adults and those with mobility issues.

I had a patient's home that flood and they couldn't get any assistance and she was going and standing in line for six, seven hours at a time. And if you got out of line, you lost your place, you had to start all over. She was wore out from just doing that because they didn't even have chairs for them to sit or anything (D4).

Participants identified that much of the information about available disaster preparedness and response resources was not communicated in a way that was relevant or easily available to their clients. Most agencies and organizations leverage social media, smartphone applications, and online methods of communication for mass rapid dissemination of information. While useful, these channels were not accessible to all aging adults, especially those without internet or mobile devices. The consensus among participants was that uneven access to disaster response resources led to prolonged and difficult disaster recovery for vulnerable older adults.

3) *Socioeconomic factors as a determinant of recovery.*

According to study participants, the ability to prepare for and recover from a disaster, as well as the speed at which these processes can happen, were influenced by socioeconomic factors such as education level and income. Many patients served by the providers in this study live on the fixed incomes offered by government assistance programs such as social security and disability. One participant noted some of the patients they see have low literacy, making it challenging to apply for disaster recovery assistance.

The other thing I wanted to say is our elderly population here, a large percent do not write. They don't write. They don't know how to write because of their education level … Or read. So the FEMA program has to have people that can understand that to help these people (E4).

Personally, I have a family member who had, so two family members. One of his brothers was very educated, understood the system, worked hard with the insurances, played hardball with them and got his house rebuilt probably within a year, was completely rebuilt while his younger nephew was in the poverty level, didn't really complete more than grade school... and his house is still not built till today … So it's interesting how education level really helps get you through the systems of insurance and the red tape (E2).

Participants shared that inadequate financial resources were a barrier to preparing for the storms. Preparedness supplies were described as a luxury, as many patients had limited margin to purchase supplies or resources needed to adequately prepare for the disaster.

That everybody is really just one paycheck away from devastation. So when something of this magnitude hits their lifestyle, it can drive them and send them over the edge to great proportions (E2).

And so to say, do you have an emergency fund? Do you have extra supplies put by? Well, no, I need these on a day to day basis (B4).

4) *Role of family in caregiving.*

Family served a source of support central to older adults’ ability to prepare for and recover from the hurricanes, according to participants. Whether it was assisting with evacuation efforts, wound care after the disaster, or providing emotional support, the formal and informal caregiving in various forms provided by family increased patient wellbeing during and after the disaster. Participants cited a multi-generational scaffolding of family support; older adults and younger family members benefitted mutually.

Having four generations in one household lends to that help that they need emotionally because they can say, "Look, I've been here for 50 years. I survived 50 storms. It's just materials we can build. We've got each other." (E2).

And here in <location redacted>, it's a cultural difference from where we're from up North. We have two generations, three generations in one home (E5).

Overall, there was a general agreement among participants that the presence of family members was a significant factor that determined whether older patients were able to adequately prepare for, evacuate from, and recover from disasters.

If you don't have family that lives somewhere and a lot of these people don't, they don't have the resources to pay for [evacuation] (D4).

“What am I going to do? Where am I going to stay? I can't afford a hotel; I don't have any family anywhere else. This is where all my family is at” (E2).

Family members also supported home care providers with formal caregiving in circumstances where providers were physically unable to reach them due to flooding or other hurricane-related transportation barriers. Providers relied on family members to facilitate patient-provider communication, continuation of care, and compliance with essential medications.

[We told patients, we’ll] write down your wound care instructions for you because your family is going to have to do it wherever you're taken to (B4).

I asked her who she would be living with, her sister. I needed that phone number; I had her phone number and then I got the daughter's phone number and the location where the daughter was at. So we can try to keep ... At least have more than one phone number to locate somebody (D4).

5) *Equating preparedness with previous hurricane experience*

Participants described that some patients took less action to plan for their health needs during disasters. Participants highlighted the tension between their patients’ desire to remain autonomous and age independently and their need for health, social and community resources, especially after the hurricanes.

For the patients that we see … they have lived in Texas for all of their whole, entire life for most of them. And their strength and everything was so pronounced prior to the storm that's going to be, and nothing can get us kind of attitude (B5).

I always tell people, "Please leave before the mandatory evacuation," because when they're sick, it's truly too late for them to leave. (D2)

Participants described that their patients cited that since past hurricane experiences did not result in serious damages, which would then negatively affect their motivation to take preparedness actions. According to participants, patients failed to account for the severity of recent storms.

I think it's that 500 year storm mentality. It won't happen another 500 years and what? We had two here within … The last two years (D3).

Both participants and patients who had lived through many hurricanes did not anticipate the strength of Hurricanes Irma and Harvey and were unprepared for the enormous amount of damage that occurred. For many patients, participants described how the unexpected strength of the storm paired with the feeling that they were well-prepared for having lived through so many previous hurricanes led to an overall decrease in actual preparedness.

I think that we've lived here so long, we're used to those hurricanes … So that's what happened, a lax … attitude of, "We've lived through all of them” (D2).

No one expected that kind of flood. I'm 56 years old. I've never seen it. I've never ... I've lived here my whole life (D2).

6) *Institutional and systemic barriers to preparedness*

Participants noted that it was not just patient attitudes and opinions that limited preparation for disasters. They identified systemic barriers to preparation as well. Participants described issues with insurance companies denying requests for extra medication and supplies needed to sufficiently bridge patients in the event of pharmacy closure or dysfunction during the disaster.

But that was a real problem and also insurance being what it is, they won't let you refill your prescription until five days before it's empty. And so people can't prepare (B4).

Insurance is not going to pay you to have a little packet, they're not going to give you extra wound care supplies, they're not going to give you extra medicine, they're not going to prepare you for that (B2).

In addition to insurance companies, participants also identified systemic problems in the organization of home-based care. Bureaucratic regulations result in a shortage of supplies and funds for their patient population, putting strain on an agency when they have to provide extras or when they run out and need to allocate accordingly.

So usually what happens is traditional Medicare people, their supplies are part of the payment that we get. But any other insurance or Medicare HMO, we set them up with a supply company and that is paid for separately. We don't supply them. But in this instance, it's either supply them or they won't have anything. So we supplied people but that was expensive (B6).

These systemic barriers not only contributed to patients’ and providers’ inability to adequately prepare for the disasters, but also had implications for home health agencies *after* disasters.

7) *Loss of material possessions represented loss of identity.*

Participants described patient stress from the loss of property and material possessions. They noted that the loss of possessions that held sentimental value, such as pictures or family heirlooms, represented the diminishment or disappearance of a sense of belonging. Participants also described patients who did not evacuate because their identity was intertwined with their material possessions. For those patients who did evacuate, participants detailed their devastation upon returning home and surveying the storm’s damage to their houses and belongings.

And when you walk in afterwards and you see who've been displaced from their home that they've had for 40 years, everything, all of their Elvis figurines, all of their elephants with the trunks up, are all gone … So a lot of these people ended up losing their furniture, belongings that they had in their families for a very long time. Things that were really important to them (B5).

The feeling of defeat of living in a one bedroom apartment compares to that three bedroom house that they've had for 40 years is absolutely the defeat and the psychological ‘why live’ anymore. And when you get to be older it is a why live anymore kind of feeling and how can you help them with that (B5).

But they'll still tell you to this day, they're not going to leave their homes, because I feel that's ... They feel that's all they have and that's where they're going to stay (D4).

8) *Disaster-related mental health care needs.*

Disaster-related trauma and stress were evident to participants in both their patients and family caregivers. Participants attributed to stress from the disaster a decline in health for some providers’ patients, including worsening symptoms of dementia and other memory impairment and chronic disease exacerbations.

But his wife actually declined quite a bit after the storm from her dementia, just because it was a traumatic experience for her (A4).

I had one patient that had a stroke after the tornado came because of the stress was just too high (E3).

And I think it's stresses those elderly patients to a point where it's almost unmanageable for them, especially the people that have no real resources and there's just so much. I mean, it was everybody, everybody (D2).

Providers also described needing additional training and resources to meet mental health needs, which they described as a barrier to supporting patients.

I didn't realize how emotional it was going to be to see those patients after they've lost lots of things and are with different people and how emotional they were. They were traumatized and so when we would see them, you'd have to be kind of, okay, let me put on a different hat. Let me think. My psychological hat or whatever, because your visit with different definitely, not just because they were somewhere else and they didn't have their supplies, but mentally the patient was different and so our visits were different that way too. I noticed that. (E5)

## Discussion

This analysis of the experiences of home-based care providers during hurricanes Harvey and Irma revealed broader themes affecting older adults that can be used to develop strategies for facilitating healthy aging and aging in place through disasters. The findings can also inform disaster mitigation, preparedness, response and recovery initiatives tailored to older adults. Our results are discussed through the lens of the Social Ecological Model of Disaster Resilience (Fig. 1).

At the individual level, the study findings identify the importance of the concept of “home,” and the associated loss of identity accompanied by infrastructure damage and loss of belongings. Home, with its familiar possessions, represents a place of safety, and therefore provides familiarity and comfort that is essential to the healthy aging process [23]. The need to keep the structural and functional integrity of older adults’ homes and possessions intact during disasters may in turn help to preserve identity, which can lead to increased levels of self-sufficiency, self-confidence, and well-being throughout the aging process. This may be especially important for those older adults living with dementia and other memory impairments.

Past efforts to support older adults and aging in place have identified gaps around preparedness actions, such as supporting planning around medication management, lack of transportation, and other specific health needs [6]. Other work has focused on messaging to inform older adults of the need to evacuate, or in preparing shelters to support older adults [24]. Policymakers and researchers must consider older adults’ strong desire to remain at home and in their home community, coupled with concerns about loss of independence and the stressors associated with economic hardship caused by the disaster. As our findings show, decision-making around evacuation was a focus among the patients served by the participants, which merits the need for innovations in ensuring the health and well-being of older adults sheltering in-place during severe disasters.

Increased support in the form of mental health interventions and community services are needed, especially with the disruption of normal routines, or when evacuation is necessary. Increasing mental health services as part of preparedness planning may mitigate potential effects attributed to the loss of independence that older adults may feel, as well as support them in taking preparedness actions. Finally, it is critical to center older adults as key stakeholders [25] in disaster-related decision-making, including focusing on preparedness education, evacuation options, and even on safety when sheltering in place.

At the interpersonal level, participants described the importance of the family in the caregiving role as both a practical extension of the home-based care provider as well as an integral part of the emotional and logistical support needed throughout the disaster cycle. Family members and other informal caregivers were seen as key components of the individual’s social support network, one that is vital to maintaining the health and function of older adults.

In disaster planning, using these networks to enable and encourage preparedness action is an important focus. Participants noted that while many patients equated preparedness with previous hurricane experience, they did not take actions to prepare due to a variety of reasons. Social support networks, with family members, informal caregivers, and home-based care providers in the forefront, can be employed to provide preparedness resources and help older adults take the steps necessary to preserve health during disasters. Educating and preparing existing community-based organizations involved in disaster response and aging to work with these networks can improve the level supports around disaster readiness for older adults.

At the community level, participants described community-level resources as the most effective and useful sources of support for their patients. Providers felt that schools, churches, and neighbors were more reliable than governmental agencies. Local emergency management, first response or public health agencies were mentioned little, if at all. Community networks, both formal and informal, functioned as a social safety net for older adults, where networks of families, faith-based organizations and neighborhoods provided disaster relief tailored specifically to the needs of the community. Future efforts to improve responses throughout the disaster management cycle around healthy aging must consider the apparent relative value of these informal and formal networks of community support into account. Stakeholders in this field, such as local response and voluntary organizations active in disasters (VOADs), can leverage the reliance and value older adults place on community-level support networks by boosting organizations and neighborhood alliances that are already embedded within the community and incorporating them into disaster planning. Increased coordination efforts between these community leaders, VOADs, and the local, state, and federal response are needed in order to integrate organized planning systems that center the health and safety of older adults.

At the societal level, it is well known that disasters disproportionately affect those who are structurally marginalized, which in the context of this study were those in rural areas, those who had lower average household income and education level, and those living with disability or chronic disease [4, 26–28]. Many fell into several of these categories. Exacerbated inequities and increased stratification exist in communities along lines of age, class, and—although not specifically stated in participants’ responses but implied due to socio-historic context—race [27]. As stated in our findings, many times home health agencies are not adequately equipped to address these disparities due to insufficient funding and limited scope of practice. Older adults may rely on federal aid programs such as social security and disability, with a limited and regulated income. These social challenges have downstream health implications, such as having to decide between paying for essential medication versus other urgent expenses, or taking on financial stress that may result in disease exacerbation. The advancing effects of climate change, which contribute to more severe and frequent disasters, will continue these disproportionate effects on the most under-resourced [29–31]. Structural inequities related to all phases of the disaster management cycle (mitigation, preparedness, response and recovery) at the societal level must be critically examined, and innovative solutions must be created in order to ameliorate these systemic issues.

At the policy level, this study demonstrated that the function, efficacy, and coordination of disaster assistance programs are often not easily accessible to those who are most in need of them. Past research has documented inequalities associated with receipt of federal disaster assistance and race [27]. This study found that the often overly-complex applications for federal assistance burdened patients with issues such as lower education levels, transportation issues, and lower technological savvy. Indeed, FEMA has committed in recent strategic planning to reduce the complexity of the organization, however numerous challenges remain [32]. Obtaining disaster assistance must be made more accessible for all older adults through changes to the complex design of these applications. Further, insurance companies share some of these same complexities. Amending policies around providing extra medications or equipment or refilling lost prescriptions so that older adults could prepare for disasters could provide support in times of emergencies. Insurers need to adapt or develop alternative plans in the event of a disaster so that older adults can adequately prepare to maintain health and medication regimens.

## Limitations

Our study has limitations that prevent these findings from being generalizable to all home-based care providers in disaster settings. The small sample size of both participants and disasters is one of these factors; while hearing from 25 participants across two hurricanes provides valuable insight on caregiving experiences within certain communities, these data present too narrow a scope to be applicable to communities outside similar demographic lines or across various kinds of disasters. Similarly, the majority of participants were white women, meaning valuable perspectives on disaster healthcare from home-based care providers of color and those identifying as men were largely excluded. In order to gain a more holistic understanding of aging and home-based care in the context of disasters, recruitment strategies must focus on including providers from varying demographic backgrounds, with a focus on prioritizing from those from traditionally marginalized groups including Black, Indigenous, and other providers of color. Future efforts to study home-based strategies that remove healthcare barriers for older adults during disasters must not only diversify recruitment to include these providers’ perspectives, but also expand the type and scope of disasters from solely studying hurricanes to analyzing a wider range including other large-scale storms, wildfires, and wide-spread infections. However, our work, one of the few to systematically consider the perspective of home-based care providers, gives valuable insights for these future studies.

## Conclusion

Health effects from disasters persist well into the recovery period, lasting months and even years. It is imperative for policy makers, representatives, and local leaders to prepare for, and respond to, the disaster-associated factors that prevent healthy aging. In this study, we conducted focus groups with home-based care providers who were responsible for the health of older adults during two large-scale hurricanes, thereby illuminating was is needed to develop strategies that support healthy aging in place in disasters. With interventions targeted towards their specific needs, older adults will be better equipped to maintain healthy levels of function and aging in-place measures, leading to better quality of life throughout the aging process.

## Supplementary Information

**Additional file 1.** Focus Group Interview Guide

## Data Availability

The datasets generated and/or analyzed during this current study are not publicly available due to ongoing use of data set but are available from the corresponding author on reasonable request.

## Abbreviations

SEMDR: Social Ecological Model of Disaster Resilience
FEMA: Federal Emergency Management Agency
HHA: Home health agencies
RN: Registered Nurses
VOADs: Voluntary organizations active in disasters
