# Supplementary material for: “Helping fill that gap:” a qualitative study of aging in place after disaster through the lens of home-based care providers
Source: BMC Geriatr. 2021 Apr 8;21:235. doi: 10.1186/s12877-021-02159-0 (PMC8033697; doi:10.1186/s12877-021-02159-0)
Supplement: Supplementary file 1 — Additional file 1. Focus Group Interview Guide [file 12877_2021_2159_MOESM1_ESM.doc]

**Focus Group Interview Guide**

**Introductory questions**

1. I am going to give you a couple of minutes to think about your experience of providing care to older adults after the recent (name of disaster) disaster, particularly among patients who were admitted to the hospital. Now think about the ones who you were surprised by—who were admitted to the hospital after the disaster, because of the disaster. Is anyone willing to share his or her experience?
2. Can you tell me the background behind why person was admitted to the hospital?
3. What types of strategies did these patients employ or not employ in terms of self-care (e.g. arranging for additional family support, ensuring adequate food in the home, etc)? If they did not practice any, why not?
4. Was there a specific trigger that led to the admission connected with the disaster that you are aware of? For example, loss of power or transportation.
5. Were there new barriers patients faced right after the disaster? What were they?
6. What other non-hospital modes of support could have been provided to this patient to avoid admission? Were they available at the time?

**Next Questions:**

1. Can you think of a patient who would have had to go to the hospital after the disaster, had you not done something unusual? What did you do?
2. Can you think of an example where a specific intervention wasn’t given or performed that could have prevented a hospitalization?
3. What are some examples of how the care you provided was different or extraordinary given the disaster?

**General questions:**

1. What has been different about the needs of your patients after the disaster versus before?
2. What were you unprepared for? What were your patients unprepared for?
3. What were you and your patients prepared for?
4. What do you wish you had known in advance?
5. What kinds of support did your patients need after the disaster?
6. Do you think that the patients we discussed are representative of your typical patient population?
7. What are your thoughts about using a toolkit that helps to support older adults health during and after disasters?

**Concluding questions:**

1. Is there anything I didn’t ask about that you think is important that would help inform this study?
2. Do you have any questions about the interview or study?
